# Acceleration-speed profiles in LaLiga: the influence of initial running speed and differences between positional roles in elite football players

**DOI:** 10.5114/biolsport.2025.150035

**Published:** 2025-04-28

**Authors:** Fabio Nevado Garrosa, Javier Mallo Saiz, José Luis Quintero-Illera, Jorge Rubio-López, Roberto López-Del Campo, Sergio Jiménez-Rubio

**Affiliations:** 1 Mediacoach Research Area, LALIGA, Madrid, Spain; 2 Faculty of Physical Activity and Sport Sciences, Universidad Politécnica de Madrid, Spain; 3 Education Faculty, Autonomous University of Madrid, Madrid, Spain; 4 School of Sport Sciences, Universidad Europea de Madrid, Madrid, Spain; 5 Sports Science Research Studies. Universidad Rey Juan Carlos, Fuenlabrada, Madrid, Spain

**Keywords:** Match analysis, Performance, Soccer, Positional roles, Initial velocity

## Abstract

The aim of this study was to examine the accelerations carried out by professional football players during an elite standard competition in relation to the initial running speed. An additional purpose was to determine the acceleration-speed (AS_0_) profile of the players in relation to the position in the team formation. Methods: A total of 8426 match observations from 363 players, categorized into six positional roles, who took part in the Spanish First Division (LaLiga) were examined with a semi-automatic multiple-camera video system during three consecutive seasons (from 2020–21 to 2022–23). The value of every acceleration performed during the games was related to its initial running speed, and the individual maximal and mean AS_0_ profiles of all the players were calculated using linear regression models. Results: A strong inverse relationship was detected between the acceleration values and the initial running speed for both the MaxAS_0_ and the MeanAS_0_ profiles (r = -0.990; p < 0.001 and r = 0.946; p < 0.001, respectively). This trend was consistent across all playing positions, with forwards exhibiting the greatest acceleration capacity and central defenders the smallest. There were differences (p < 0.05–0.001) in the slope of the AS_0_ profile between playing positions. Conclusions: This study shows that traditional fixed acceleration thresholds might overestimate high-intensity efforts that begin at low movement speeds and underestimate those that start at higher speeds. Thus, AS_0_ profiles can provide a valuable tool to monitor physical match performance more objectively and help to discriminate the acceleration capacity between playing positions.

## INTRODUCTION

Football is an intermittent sport characterized by a predominance of low-intensity activities interspersed with short high-intensity efforts [1]. The traditional approach to match analysis has consisted in the study of distances covered by players in pre- established fixed speed zones, which has allowed the elaboration of detailed positional profiles [2]. Specifically, distances covered at high velocities and sprinting have been identified as valuable variables to discriminate between footballers from different competitive standards [3]. However, many short intense actions which do not exceed the high velocity threshold as accelerations, decelerations or changes of direction have not been taken into account [4–6] despite being mechanically more demanding than running at constant speeds [7–8]. These activities are essential to evaluate match performance [9] and are related to the development of neuromuscular fatigue [10] and might lead to an increase in the risk of sustaining injuries [11].

Acceleration, from a physics point of view, is defined as the variation of speed per unit of time. Hence, the faster a player is moving during a match, the smaller is his capacity to accelerate. This has a practical implication, as when a player is running or sprinting, his possibilities to increase the acceleration are severely compromised [12]. On the other hand, the maximal peaks of acceleration are reached at the beginning of the movements or when moving at low speeds. For many years, studies of accelerations in team sports have overlooked the starting speed, carrying the same methodological bias as with speed, that is, using established fixed categories based on absolute thresholds. Under this approach, high-intensity accelerations have been considered in different studies as those exceeding a range between 2.5 and 4 m · s^−2^, with 3 m · s^−2^ being the most frequent threshold [6, 13–16]. Nevertheless, accelerations should be related to the initial running speed to avoid underestimating all the high demanding metabolic events which do not reach the selected threshold or overestimating the importance of those accelerations that begin at a low initial running speed or from a standing position.

In recent years, a new methodological approach to evaluate the intensity of actions from an individual perspective has been developed [12]. These authors calculated the maximal acceleration of football players in relation to different initial speeds, from which they defined high-intensity accelerations as those that exceeded 75% of the maximal acceleration for each locomotor category. Building on this, different studies have examined the acceleration-speed (AS_0_) profile of football players, providing valuable information about the acceleration capacity at different movement velocities [17–21].

From a practical point of view, the AS_0_ profile is a conceptual evolution from the force-velocity profile [22–24] that has been applied to monitor the sprinting performance of football players. In addition to providing a more comprehensive understanding of acceleration requirements in all the velocity spectrum, one of the main advantages of the AS_0_ profile is that it can be calculated from training or match data, without requiring specific fitness tests [19]. Recent studies have utilized this approach to examine the variability of the AS_0_ profile in football players in relation to their playing position, the day of the microcycle or during different phases of the season [25, 26].

However, to the best of our knowledge, all the studies published so far have calculated the AS_0_ profile based on data obtained from Global Positioning System (GPS) devices. Therefore, they have included a limited number of subjects in the observations, as the players examined belonged to a single team [11, 17, 18]. In recent years, the most important competitions in Europe have used semi-automatic optical systems to track the movements of players during the games [27, 28]. These systems are a valuable tool to increase game insight, as all the players taking part in the match can be non-invasively monitored to provide a detailed evaluation of their match performance [29, 30]. Thus, the inter-individual differences in the AS_0_ profile between players from different teams of the same league and the effect that playing position might have on these variables remain uncertain. Furthermore, a comprehensive analysis of the AS_0_ profiles within the competition will provide the necessary knowledge to establish, in the future, acceleration thresholds by positional role across different ranges of initial speed. This approach would enable a more rigorous and effective analysis of the competitive acceleration demands compared to the current method, which relies solely on an absolute threshold regardless of initial speed. [11].

The aim of this study was to examine the accelerations carried out by professional football players during an elite standard competition in relation to the initial running speed. An additional purpose was to determine the AS_0_ profile of the players in relation to different playing positions in the team formation.

## MATERIALS AND METHODS

### Experimental approach to the problem

A retrospective, descriptive longitudinal design was applied to analyse the acceleration efforts of football players during three consecutive seasons (from 2020–21 to 2022–23) from the Spanish First División (LaLiga). The study included all the 1140 matches played during this period. All data were provided by LaLiga with full authorization and the necessary permission granted to use the data for the purposes of conducting this research and subsequently publishing the findings.

### Participants

Only the players who played a minimum of 90 minutes in the match were considered for analysis [31], leading to a total of 8426 match observations from 363 players. These players were further classified by the Mediacoach system into six groups according to their playing position [8, 31]: central defenders, CD (n = 73; observations = 2601); full backs or external defenders, FB (n = 70; observations = 1919); central midfielders, CM (n = 57; observations = 1372); offensive midfielders, OM (n = 40; observations = 745), wingers or external midfielders, WG (n = 73; observations = 873); forwards, FW (n = 50; observations = 916). The average number of observations per player by season (from 2020–21 to 2022–23) was 13.1, 12.5 and 12.2, respectively. When a player changed his position during a game, it was assigned automatically considering the position in which the player had spent the greater amount of time during the match. Goalkeepers were excluded from the analysis as their activity profile is very different to the other game positions. The study received ethical approval from the Universidad Politécnica de Madrid based on the latest version of the Declaration of Helsinki (Ref.: 489/24022020).

### Procedures and data analysis

Physical match performance of the players during the games was determined using the Mediacoach system, which is based on a series of super 4K-HDR cameras that track the movements of the players at a frequency of 25 Hz (LaLiga, Madrid, Spain). This semi-automatic multiple-camera video technology (VID) is integrated in a positioning system (Tracab-ChyronHego VID) to calculate the position (x,y coordinates) of the 22 players on the field throughout the whole match. The Mediacoach system incorporates data correction functionalities for each player to ensure the accuracy of the data collected. In addition, previous studies [30, 32] have proven the validity and reliability of this instrumentation for match analysis.

### Contextual factors and variables

The individual AS_0_ profile of the players, classified according to their positional role, was calculated for every match observation that was included in the investigation [19]. This dataset related each acceleration performed by the players during the match to its initial running speed (IniS). The data were filtered and processed using the “density-based clustering algorithm” (DBSCAN) [33], which made it possible to remove the atypical or outlying values for each player and construct a scatter plot, in which the x-axis was the speed (km · h^−1^) and the y-axis the acceleration (m · s^−2^). The two maximal values of acceleration (MaxAcc) performed for each 0.72 km · h^−1^ subinterval from all the players of the same positional role were selected for further analysis [19]. Using a linear regression model, the maximal AS_0_ profile (MaxAS_0_) for each playing position was determined [19]. The same procedure was followed to calculate the mean AS_0_ profile (MeanAS_0_) for each playing position and to calculate 75% of this value (Mean75AS_0_). In this case, the two maximal values for accelerations of every player for each subinterval were identified and fitted with a linear regression model. To characterize each AS_0_ profile, the following variables were calculated [19]:

– – A_0_: maximal theoretical acceleration (interception of the regression line with the y-axis; speed: 0 km · h^−1^)
– S_0_: maximal theoretical running speed (interception of the regression line with the x-axis; acceleration: 0 m · s^−2^)
– Slope (rate of change of the acceleration): AS_0_slope = -A_0_/S_0_

### Statistical analysis

Pearson’s correlation coefficients were utilized to assess selected bivariate relationships, along with their respective 95% confidence intervals. Correlation coefficients (r) of 0.3, 0.5, 0.7, and 0.9 were categorized as indicating low, moderate, high, and very high relationships, respectively. Regression analyses were conducted to examine the relationships between the initial running speed and the maximal accelerations, both for the entire sample and for each specific playing position. Differences in intercepts and slopes were analysed using ANOVA tests for between-subject effects. RStudio (version: 2024.09.0+375) was used to edit worksheets, manipulate data, and create graphs. Statistical significance was determined at an alpha level of p ≤ 0.05. IBM SPSS Statistics version 29.0.2.0 software for Mac (SPSS Inc., IL, USA) was used for statistical analysis.

### RESULTS

The accelerations performed by the players during the games showed a very high correlation with the initial running speed of the movements. The reverse relationships, both in the MaxAS_0_ and MeanAS_0_ profile (r = -0.990; p < 0.001 and r = -0.946; p < 0.001, respectively), were consistent across all the playing positions, as can be seen in Table 1.

**TABLE 1:** Relationships between maximal acceleration and initial running speed in the maximal and mean acceleration-speed profiles of professional football players from different playing positions.

	Playing Position	Pearson’s r IniS vs AccMax	p-Value	95 % CI
Max AS_0_	All	-0.990	< 0.001	-0.991– -0.987
	Central Defender	-0.993	< 0.001	-0.996– -0.990
	Full Back	-0.996	< 0.001	-0.998– -0.994
	Central Midfielder	-0.993	< 0.001	-0.996– -0.989
	Offensive Midfielder	-0.989	< 0.001	-0.993– -0.982
	Winger	-0.992	< 0.001	-0.994– -0.987
	Forward	-0.993	< 0.001	-0.996– -0.989

Mean AS_0_	All	-0.946	< 0.001	-0.948– -0.945
	Central Defender	-0.951	< 0.001	-0.954– -0.948
	Full Back	-0.950	< 0.001	-0.953– -0.947
	Central Midfielder	-0.954	< 0.001	-0.957– -0.951
	Offensive Midfielder	-0.961	< 0.001	-0.964– -0.957
	Winger	-0.931	< 0.001	-0.935– -0.927
	Forward	-0.949	< 0.001	-0.952– -0.945

**FIG. 1:**
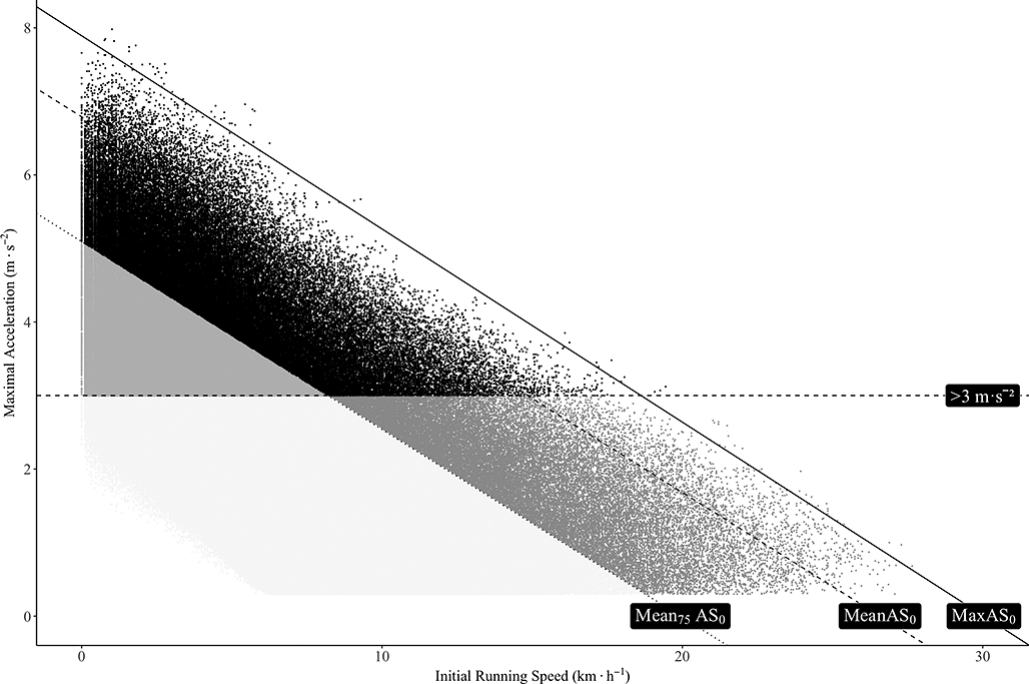
Relationship between maximal acceleration and initial running speed of professional football players.

**FIG. 2:**
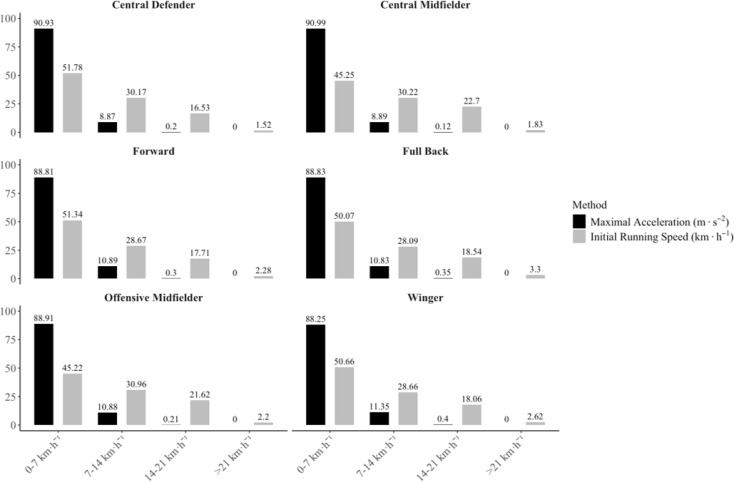
Percentage of accelerations carried out by professional football players from different playing positions in relation to the initial running speed.

The relationship between MaxAcc and IniS for all the players is presented in Figure 1. As can be observed in this figure, there are many demanding efforts that do not reach the traditional high-intensity acceleration threshold set at 3 m · s^−2^. In practical terms and irrespective of the playing positions, between 88 and 91% of all the accelerations exceeding 3 m · s^−2^ carried out by the players were performed at IniS below 7 km · h^−1^ (Figure 2).

**FIG. 3:**
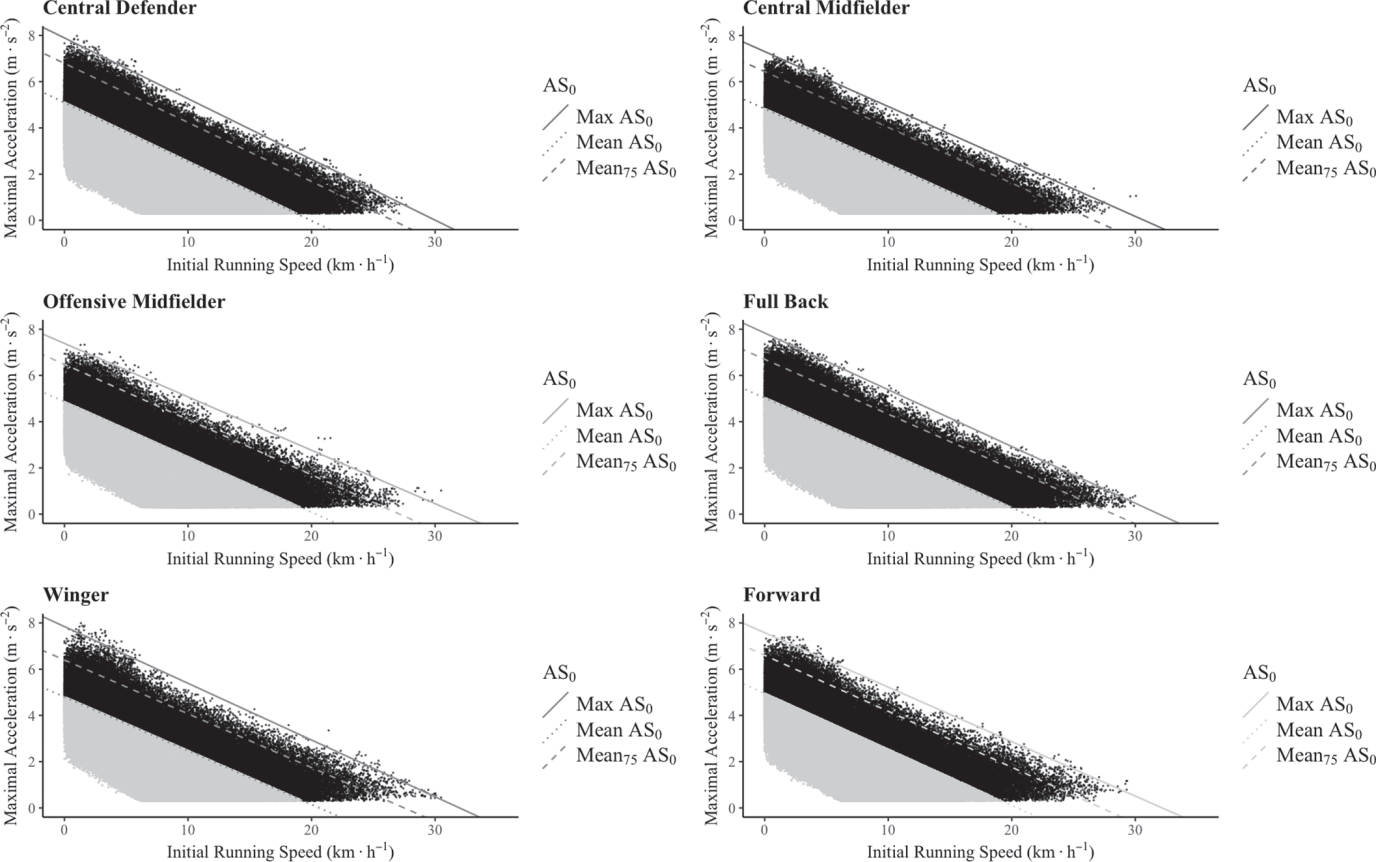
Acceleration-Speed profiles of professional football players according to their playing position. MaxAS_0_: Maximal AS_0_ profile; MeanAS_0_: Mean AS_0_ profile, Mean_75_AS_0_: 75% of Mean AS.

Figure 3 shows the MaxAS_0_ and MeanAS_0_ profiles for the players according to their playing position. The regression analysis model revealed that IniS explained 97.7% and 89.5% of MaxAcc when considering the MaxAS_0_ and MeanAS_0_ profile, respectively, of all the players. When the playing positions were studied in isolation, IniS explained between 97.7 and 99.3% of the acceleration values in the MaxAS_0_ profile, whereas IniS explained between 86.8 and 95.0% of the acceleration values in the profile MeanAS_0_ (Table 2). There were no significant differences in the slope of the different AS_0_ profiles.

**TABLE 2:** Regression analysis model of the maximal and mean acceleration-speed profiles of professional football players from different playing positions.

Position	Max AS_0_					Mean AS_0_					Differences	

	Equation	R^2^	P-value	95% Confidence Interval		Equation	R^2^	P-value	95% Confidence Interval		Intercept	Slope
	
				Intercept	Slope				Intercept	Slope	p-Value	p-Value
All	7.630 -0.242 (IniS)	0.979	< .001	7.577–7.683	-0.245– -0.239	6.577 -0.241 (IniS)	0.895	< .001	6.562–6.593	-0.242– -0.240	< 0.001	n.s.d.

Central Defender	7.892 -0.263 (IniS)	0.987	< .001	7.781–8.003	-0.270– -0.256	6.790 -0.255 (IniS)	0.905	< .001	6.757–6.824	-0.258– -0.253	< 0.001	n.s.d.

Full Back	7.838 -0.246 (IniS)	0.993	< .001	7.757–7.920	-0.250– -0.241	6.698 -0.237 (IniS)	0.950	< .001	6. 663–6.732	-0.240– -0.235	< 0.001	n.s.d.

Central Midfielder	7.304 -0.238 (IniS)	0.987	< .001	7.201–7.408	-0.244– -0.231	6.435 -0.241 (IniS)	0.911	< .001	6.399–6.471	-0.244– -0.239	< 0.001	n.s.d.

Offensive Midfielder	7.385 -0.231 (IniS)	0.977	< .001	7.251–7.519	-0.239– -0.223	6.478 -0.239 (IniS)	0.923	< .001	6.436–6.520	-0.242– -0.236	< 0.001	n.s.d.

Winger	7.843 -0.245 (IniS)	0.983	< .001	7.719–7.966	-0.252– -0.238	6.396 -0.233 (IniS)	0.868	< .001	6.357–6.435	-0.235– -0.230	< 0.001	n.s.d.

Forward	7.579 -0.236 (IniS)	0.987	< .001	7.475–7.683	-0.242– -0.230	6.599 -0.242 (IniS)	0.900	< .001	6.558–6.639	-0.245– -0.239	< 0.001	n.s.d.

Table 3 shows the differences between playing positions regarding their maximal theoretical acceleration. The A_0_ value of the MaxAS_0_ profile of the forwards showed greater significant differences in relation to the other playing positions. In relation to the MeanAS_0_ profile, there were significant differences (p < 0.001) between central defenders, fullbacks and forwards and all the other positions.

**TABLE 3:** Differences between playing positions in the theoretical maximal acceleration values determined for the maximal and mean acceleration-speed profiles.

**Max AS**_0_					
	**Full Back**	**Central Midfielder**	**Offensive Midfielder**	**Winger**	**Forward**
Central Defender	n.s.d.	< 0.001	< 0.001	n.s.d.	< 0.001
Full Back		< 0.001	< 0.001	n.s.d.	< 0.001
Central Midfielder			n.s.d.	< 0.001	< 0.001
Offensive Midfielder				< 0.001	< 0.05
Winger					< 0.001

**Mean AS_0_**					
	**Full Back**	**Central Midfielder**	**Offen sive Midfielder**	**Winger**	**Forward**
Central Defender	< 0.001	< 0.001	< 0.001	< 0.001	< 0.001
Full Back		< 0.001	< 0.001	< 0.001	< 0.001
Central Midfielder			n.s.d.	n.s.d.	< 0.001
Offensive Midfielder				< 0.05	< 0.001
Winger					< 0.001

The differences between playing positions in the rate of change of acceleration (slope) of the MaxAS_0_ and MeanAS_0_ profile are summarized in Table 4. The slope of the MaxAS_0_ and MeanAS_0_ profile of the central defenders was significantly different (p < 0.001) from all the other positional roles.

**TABLE 4:** Differences between playing positions in the slope of the maximal and mean acceleration-speed profile of professional football players.

**Max AS_0_**					
	**Full Back**	**Central Midfielder**	**Offensive Midfielder**	**Winger**	**Forward**
Central Defender	< 0.001	< 0.001	< 0.001	< 0.001	< 0.001
Full Back		< 0.05	< 0.05	n.s.d.	< 0.05
Central Midfielder			n.s.d.	n.s.d.	n.s.d.
Offensive Midfielder				< 0.05	n.s.d.
Winger					< 0.05

**Mean AS_0_**					
	**Full Back**	**Central Midfielder**	**Offensive Midfielder**	**Winger**	**Forward**
Central Defender	< 0.001	< 0.001	< 0.001	< 0.001	< 0.001
Full Back		< 0.05	n.s.d.	< 0.05	< 0.05
Central Midfielder			n.s.d.	< 0.001	n.s.d.
Offensive Midfielder				< 0.05	n.s.d.
Winger					< 0.001

## DISCUSSION

The aim of this study was to examine the accelerations performed by elite professional football players during competitive matches in relation to the initial running speed. The findings indicate that the acceleration capacity is strongly influenced by the initial speed of the movement, regardless of the positional role of the players in the team formation. The implementation of acceleration thresholds derived from the AS_0_ profile, which take into consideration initial speed, provides a more objective and precise procedure to monitor the physical demands of match play. This approach reduces the associated limitations when absolute acceleration thresholds are used, which tend to underestimate high-speed, low-acceleration efforts. In addition, the AS_0_ profile proves to be a valuable tool for discriminating performance characteristics between playing positions.

In recent years, the speed and accelerations of football players during competition have been assessed independently and classified into fixed categories [2, 13]. The quantification of accelerations has been a topic of increasing importance as they have been shown to be related to the mechanical load of the game and the associated neuromuscular fatigue [5, 8, 10]. For this purpose, many studies have established thresholds between 2.5 and 4 m · s^−2^ to characterize these high-intensity episodes. However, as can be seen in Table 1, the MaxAcc capacity of the players is dependent on the IniS of the individual actions [12]. To our knowledge, this is the first study that has determined that more than 90% of the MaxAcc values can be explained by the IniS in elite professional football players (Table 2). This finding was consistent for all playing positions, with minor differences in the strength of the correlation between positional roles. From a practical point of view, this reflects the fact that the greater the initial running speed is, the lower acceleration capacity the players have. For instance, it can be observed in Figure 1 that the great majority of accelerations above 3 m · s^−2^, probably the most common threshold employed elsewhere [2, 6, 13–16], are at the left side of the figure. From a practical point of view, this translates into 90% of the accelerations carried out at IniS below 7 km · h^−1^ (Figure 2). Hence, using fixed acceleration thresholds (i.e., 3 m · s^−2^) can lead to overestimating the intensity of the movements that start at low running speeds and underestimating those that begin at greater speeds. On the other hand, when considering the AS_0_ profile, there is a more homogeneous distribution of the intense efforts, as accelerations that exceed the threshold are related to the speed at which the movements are started.

Building on the above observations, a new procedure to monitor the intensity of accelerations should be developed in which accelerations and IniS are considered together. Ideally, from a training perspective, it would be very interesting to assess the individual acceleration capacity of each player to identify the individual thresholds and be able to monitor the load more precisely [34]. A recent study [35] examined the acceleration capacity of players in relation to their maximal values. However, the observations carried out by these authors were restricted to a single team during consecutive training microcycles. Hence, in order to examine large populations of players, as is the case in league tournaments, it is not possible to have this information, as each coaching staff has a different approach to evaluating the fitness capacity of their players. For this reason, an alternative approach could be to establish specific acceleration thresholds according to the standard of the competition and to the IniS of the movements. This could also have a valuable effect, as it would make it possible to study the inter-individual differences between players of the same competitive standard.

To bring light to this topic, in the present study we calculated the MaxAS_0_ profile for each playing position during three consecutive seasons of one the most important football competitions in the world (LaLiga). However, this maximal profile could not be representative for all the players in the competition, as it was calculated based on the subjects that reported the MaxAcc values for each IniS. For instance, we also calculated the MeanAS_0_ profile, which integrated the average values of all the players with the same positional role in the competition. In addition, and searching for a deeper interpretation of the data, we also traced parallel lines at 75% of the MeanAS_0_ profile to help develop a more objective categorization of the acceleration efforts. In this sense, as Figure 3 shows, the Mean75AS_0_ profile could be an adequate threshold to define the intensity of accelerations. Altogether, these findings have a very important practical application, as there are considerable differences between the traditional approach – which defines high-intensity accelerations as those exceeding a fixed threshold of 3 m · s^−2^ – and the alternative approach presented in the current investigation using the Mean75AS_0_ profile.

It is well known that the physical demands of elite football are highly influenced by the playing position of the footballers [36]. Thus, in this study we further investigated the differences in the AS_0_ profile between positional roles. As can be seen in Table 3, there were differences between most of the playing positions in the A_0_, both in the MaxAS_0_ and MeanAS_0_ profile. This finding is consistent with previous research that has shown positional differences regarding total distance covered [37], distances covered at different speeds [16, 38–40], accelerations and decelerations [16, 17, 20, 39] and even the types of actions carried out exceeding 85% of the maximal speed [41]. Interestingly, we did not observe differences in the A_0_ value between central midfielders and wingers in the MeanAS_0_ profile, despite these positions having different kinematic and aerobic demands [42]. However, the slope of the MeanAS_0_ was significantly different between the two roles (Table 4), which could represent a greater capacity of wingers to accelerate when running at higher speeds. There were no significant differences in the slopes of the MaxAS_0_ and MeanAS_0_ profile for all the other positional roles. This indicates that the profiles were consistent for all the positions, with differences detected only in the MaxAcc value when starting from a stationary position.

Regarding the limitations of the study, it is important to highlight that in our investigation we examined all the different teams that took part in a league tournament. Consequently, each team had a particular game model and team formation, selecting players with different physical and technical-tactical characteristics for the same positional role. It would be interesting to examine in future research whether there are specific AS_0_ profiles related to the game models adopted by the teams. Another limitation of the study was that accelerations were calculated based on video tracking technology. It remains uncertain whether the use of inertial measuring units would have reported different AS_0_ profiles for the different playing positions. Our investigations relied exclusively on data analysis, as video observations of the recorded actions would have been impractical.

## CONCLUSIONS

This study presents novel findings that had not previously been scientifically demonstrated with such a large sample of elite players. The results indicate that the IniS at which an acceleration effort is performed when playing football accounts for a substantial proportion of the MaxAcc values reached. Consequently, accelerations should not be analysed in isolation from the IniS, as it is essential to consider the velocity at which the acceleration begins. Moreover, the study revealed statistically significant differences in the AS_0_ profiles of players from different playing positions in an elite standard competition. These findings underscore the importance of respecting the positional specificity when designing and planning training programmes in football.

Based on the findings of this research, it is essential to consider the IniS of the movements when monitoring the acceleration demands during training and competition. Specific AS_0_ profiles should be developed for every playing position to analyse the physical demands more accurately. From a practical perspective, the analysis of the acceleration efforts based on absolute thresholds and without accounting for the initial speed can lead strength and conditioning coaches to draw inaccurate conclusions, as many important high-speed, low-acceleration efforts are excluded. Thus, when programming and designing the conditioning sessions, coaches should not only think of high-demanding accelerations as those starting from a static position, but also considerer those actions that begin at greater running speed despite not exceeding the traditional high-intensity acceleration threshold. In this sense, the determination of the AS_0_ profile is extremely valuable to effectively monitor the acceleration demands across the entire velocity spectrum and to help design more valid training drills in accordance with the specific demands of the competition. Additionally, the AS_0_ profile could be an additional non-invasive and objective tool to evaluate the physical performance of players during the season.
